# Pomegranate Husk Scald Browning during Storage: A Review on Factors Involved, Their Modes of Action, and Its Association to Postharvest Treatments

**DOI:** 10.3390/foods11213365

**Published:** 2022-10-26

**Authors:** Mahshad Maghoumi, Maria Luisa Amodio, Danial Fatchurrahman, Luis Cisneros-Zevallos, Giancarlo Colelli

**Affiliations:** 1 Dipartimento di Scienze Agrarie, Degli Alimenti e Dell’ambiente, Università di Foggia, Via Napoli 25, 71122 Foggia, Italy; 2 Department of Horticultural Sciences, Texas A&M University, College Station, TX 77843, USA

**Keywords:** pomegranate, browning, oxidative stress, long term storage, husk scald, polyphenol oxidase, postharvest treatments

## Abstract

The pomegranate (*Punica granatum* L.), which contains high levels of health-promoting compounds, has received much attention in recent decades. Fruit storage potential ranges from 3 to 4 months in air and from 4 to 6 months in Controlled Atmospheres (CA) with 3–5% oxygen and 10–15% carbon dioxide. Storage life is limited by decay, chilling injury, weight loss (WL), and husk scald. In particular, husk scald (HS) limits pomegranate long-term storage at favorable temperatures. HS appears as skin browning which expands from stem end towards the blossom end during handling or long-term storage (10–12 weeks) at 6–10 °C. Even though HS symptoms are limited to external appearance, it may still significantly reduce pomegranate fruit marketability. A number of postharvest treatments have been proposed to prevent husk scald, including atmospheric modifications, intermittent warming, coatings, and exposure to 1-MCP. Long-term storage may induce phenolic compounds accumulation, affect organelles membranes, and activate browning enzymes such as polyphenol oxidases (PPO) and peroxidases (POD). Due to oxidation of tannins and phenolics, scalding becomes visible. There is no complete understanding of the etiology and biochemistry of HS. This review discusses the hypothesized mechanism of HS based on recent research, its association to postharvest treatments, and their possible targets.

## 1. Introduction

One of the most well-known fruits in the world, the pomegranate (*Punica granatum* L.), is likely native from Iran and grown in areas with a Mediterranean environment [1]. The fruit is made up of an albedo, septa, membranes, a hard, leathery outer layer, and many arils, where a translucent sac that holds 80% juice and 20% seed envelops each edible aril [2]. This fruit is considered as a non-climacteric fruit [3] and is characterized by low rate of postharvest respiration and ethylene production. Pomegranate is a rich source of organic acids, micro- and macro-nutrients, as well as anti-mutagenic, anti-inflammatory, anti-hypertension, and antioxidant compounds [4], which has influenced the increase in the demand for pomegranate fruit and of their products due to the fruit’s high-valued health advantages and the public’s growing awareness of functional foods [5]. Pomegranate harvest season is in the fall and lasts for fewer than three months, although it may be kept for several months in cold storage with a high humidity level. The fruit nevertheless experiences both qualitative and quantitative losses [6].

Due to the slowing down of cell metabolism at low temperatures and the delay in senescence, refrigerated storage of fruits and vegetables allows for the preservation of their quality after harvest [7,8,9]. However, some tropical and subtropical fruit, such as pomegranates, are not ideal for cold storage preservation since it results in physiological damage.

**Figure 1:**
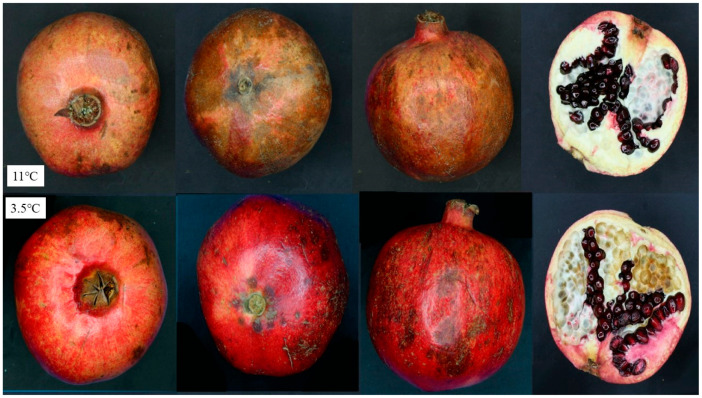
Husk scald and chilling injury in pomegranate “Wonderful” fruit skin after 120 days of storage at 11 and 3.5 °C and ≥95% RH, respectively.

The main factors that, in general, may limit pomegranate ability to be stored and accelerate their quality decline include WL, shriveling, decay, chilling injury, husk scald and decrease flavor acceptability. Fruit transpiration, that causes WL and hardening and browning of the husk, is a significant storage challenge for pomegranate fruit [10,11] while additional factors affecting pomegranate storage life include decay, that is frequently brought on by the presence of a fungal inoculum in the fruit’s blossom end [6] and this condition is worse at temperatures above 5 °C [12]. Chilling injury (CI) is a physiological disorder that takes place at low storage temperature (<5 °C) in pomegranates, and it manifests as internal discoloration of arils and albedo as well as brown spots and pitting in the skin, while on the other hand, husk scald (HS) typically originates from the stem end and manifests as a superficial browning of the skin and happens during storage temperatures above 5 °C. Differing from CI, HS does not damage the arils or the white locular septa of the fruit, and its severity can reach up to 60% of the skin [13,14,15] Figure 1.

When fruit is stored at 6–10 °C to avoid CI, the effects of superficial scald (husk scald) are typically more severe, and so it can also happen at storage temperatures above 10 °C and these scalded portions of the fruit may become prone to fungal deterioration in advanced stages [16]. Table 1 provides a summary of the differences between husk scald and chilling injury. It is assumed that CI and HS do not appear in the same fruit since they are mutually exclusive due to differences in storage temperatures, however, it has been suggested recently that both disorders can appear simultaneously at temperatures of 7 °C [17]. In general, scalding decreases the marketability of fresh pomegranate even when the internal quality remains in good conditions, because the quality of fruit is determined by both internal and external attributes [5,15,18]. It is thought that skin browning is the result of the oxidation of phenolic compounds [15,19].

Although there is a lack of information on scald disorder, the review’s goal is to address factors that impact pomegranate husk scald development, as well as the potential mode of action involved.

## 2. Evidence for Husk Scald Incidence Mechanism

Weight loss, microbiological contamination, oxidative stresses, and browning are important variables that might decrease the storage life of fruit and vegetables during cold storage and handling. Lower or mild degrees of stress tend to enhance the levels of cellular antioxidant compounds, whereas severe stress may result in a decrease in such levels and the development of postharvest physiological disorders [20,21,22]. Reactive oxygen species (ROS) are scavenged by a sophisticated antioxidant defense system in plants that include both non-enzymatic and enzymatic components. Non-enzymatic antioxidant compounds include the main cellular buffers as well as a diversity of compounds including phenolic compounds, carotenoids and alkaloids [23]. Phenolics are plant secondary metabolites that are generated and accumulate in the plant, for example, by the activation of phenylalanine ammonia-lyase (PAL) [24]. These substances could be categorized as tannins, flavonoids, hydroxycinnamate esters and lignin among others. On the other hand, superoxide dismutase (SOD), catalase (CAT), ascorbate peroxidase (APX), and other enzymes are examples of enzymatic antioxidants [25,26]. Pomegranates are rich in phenolic compounds, and various studies have suggested that oxidative stress that occurs in the fruit skin while stored at temperatures over 5 °C, may play a significant role in husk scald disorder [27]. Enzymatic browning is the second leading cause of quality loss in fruits and vegetables, after microbial infection, and the food sector has prioritized preservation against oxidation during storage and handling [28].

**Figure 2:**
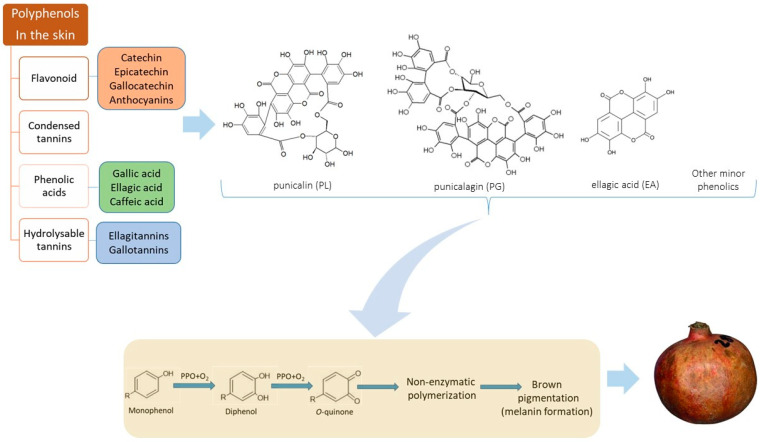
Pomegranate polyphenols in the skin, chemical structures of punicalin (PL), punicalagin (PG) and ellagic acid (EA), and brown pigment (melanin) formation from phenolic compounds [29,30,31].

Polyphenol oxidase (PPO) and peroxidase (POD) are the main enzymes that cause browning in plants. PPO is a type of enzyme known as an oxidoreductase. It catalyzes the conversion of monohydroxy phenols (e.g., phenol, tyrosine, and p-cresol) into *o*-dihydroxy phenols (e.g., catechol, dopamine, and adrenalin), as well as the dehydrogenation of *o*-dihydroxy phenols into o-quinones. Figure 2 illustrates how the formation of melanin and the oxidation of phenolic chemicals to quinones give foods their dark color [29].

Peroxidases (POD), which perform mono-electron oxidation on a wide range of composites in the presence of hydrogen peroxide, are additional enzymes which have polyphenol oxidation activity [32]. POD is a common enzyme in plants, but it still has an unclear mechanism of action. It has been suggested that the function of this enzyme depends on the availability of hydrogen [33].

As illustrated in Figure 2, the principal polyphenols in pomegranate peel are ellagitannins (hydrolysable tannins), phenolic acids (mostly hydroxybenzoic acids), and flavonoids (anthocyanins and other complex flavonoids) [34]. Punicalagin (PG) (C_48_H_28_O_30_) is the most abundant polyphenol in pomegranate skin [35], which are hexahydroxydiphenic acid (HHDP) esters coupled to polyols [36]. The PG content in the peel can reach up to 66% of the total polyphenols, it is soluble in water and can be found naturally as reversible anomers, alpha and beta; nonetheless, they are frequently referred to as punicalagin [37]. By releasing one HHDP, PG can be hydrolyzed to punicalin (PL), another ellagitannin, and both PG and PL can be hydrolyzed to Ellagic acid (EA) under particular conditions [38]. Although the structural units of PG, PL, and EA are similar (Figure 2), they differ in terms of molecular size, polarity, and solubility [30]. Moreover, flavonoids of pomegranate skin include catechin, epicatechin, quercetin, anthocyanins and procyanidins [39].

It was discovered that in scalded pomegranate fruits, PPO and POD activity increased while total phenolic and total tannin components decreased [15]. Additionally, there was a correlation between husk scald injury and the quantity of extractable *o*-dihydroxy phenols detected in the skin affected regions. Although the amount of *o*-dihydroxy phenols in pomegranate skin is quite little, it has been demonstrated that during storage, husk scald can occur as result of the enzyme-mediated oxidation of phenols present in the skin [18]. Scalded fruits had decreased antioxidant activity and total phenolic components, which was followed by a high expression ratio of PPO to PAL [17].

Temperature has a considerable impact on the rate of enzymatic browning. Fruit and vegetables do not become brown when stored at low temperature, and PPO’s enzymatic activity ceases at temperatures below 7 °C even though it is not deactivated [29].

In addition, CAT activity in pomegranate peel negatively correlated with the browning index while ascorbic acid oxidase (AAO), PPO, and POD favorably correlated with the skin browning index [19]. Accordingly, it seems that the accumulation of H_2_O_2_ with the decline of CAT activity made the tannins oxidize to browning substances. In the peel of scalded fruit, it was shown membrane instability, increased lipid peroxidation, and enhanced carotenoid production, which may suggest a response to oxidative stress [18].

Punicalin levels were strongly and negatively connected with husk scald, but punicalagin had no significant correlation to the incidence of scald. This finding raises the possibility that high punicalin levels could prevent the occurrence of HS in pomegranate fruit [40].

The symptoms and occurrence of pomegranate and apple scald are similar, however, current research indicates that the biochemical reasons and control mechanisms of the two disorders are different. Diphenylamine (DPA) treatment as an antioxidant ingredient, for instance, can decrease apple scald, but it has no effect on pomegranate scald [18,41]. Although DPA does not affect scald development in pomegranates, researchers did not eliminate the possibility that an oxidation process is involved in symptom development [18].

Anthocyanins are water-soluble polyphenolic pigments that are responsible for the appealing red-violet-blue hues of many fruits, especially pomegranate arils and husks [42]. Anthocyanins also have significant antioxidant action [43]. However, no links between anthocyanins and the signs of husk scald were discovered [40].

The findings imply that neither the total soluble solids content nor the titratable acidity or husk color are connected to the development of husk disorders [40].

The study on the anatomical changes in HS of pomegranate skin showed cracks in the epidermis and cuticle as well as cell degradation only in the very outer layers of the skin, while the inner layers seemed intact [17]. HS fruit also showed skin hardness as a result of transpiration and water loss [44]. Additionally, the monitoring of changes in tissue thickness was conducted to determine the trends of water loss on pomegranate fruit in relation to the peel tissues and location. During fruit storage, waxy cuticles fragmented more rapidly and microcracks widened. Compared to the stem end of the fruit, the calyx-end and equatorial region of the fruit exhibited a significantly higher number of lenticels, a larger lenticel size, and a generally thinner peel. The calyx end of the fruit was more susceptible to water loss than the equatorial- and stem-end regions of the fruit [45].

Gene *Pgr023188* encoding uncharacterized protein upregulated in HS fruits, this gene has 50% similarity to gene *BVC80_1667g87* that encodes for glycine rich protein (GRPs). These genes are involved in increasing tolerance during water stress [46]. Moreover, genes associated with stress (*Pgr000486* and *Pgr006284*), defense (*Pgr011016*), oxidative stress (*Pgr006284*) and Glycosyltransferase (GTs) (*Pgr007593*) which is crucial for the biosynthesis of secondary metabolites were highly presented in HS skin [44]. However, no comprehensive model of mode of action of HS development involving these genes have been reported so far in the literature.

## 3. Hypothetical Model of Pomegranate Husk Scald Incidence

**Figure 3:**
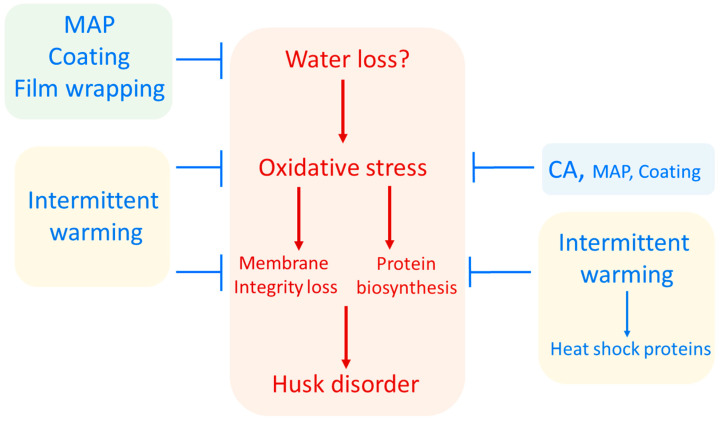
Hypothesis of pomegranate husk scald incidence during storage and the possible targets of postharvest treatments. Accordingly, MAP, coating and film wrapping systems would work mainly by preventing water loss, while CA would have a mode of action involving a decrease in oxidative stress by reducing the presence of oxygen. Furthermore, MAPs and coatings may also play in part this role of reducing oxidative stress. On the other hand, intermittent warming may exert its effects by inducing heat shock-like proteins in a hierarchical stress response model or alternatively by reducing oxidative stress and preserving membrane fluidity.

A possible mechanism of husk scald development is summarized in Figure 3. It is suggested that when water loss is present in pomegranate fruit browning of the skin is enhanced [47,48]. In addition, it has been reported that oxidative stress is induced by water stress, which leads to the synthesis of nicotinamide adenine dinucleotide phosphate (NADPH) oxidase and trigger the oxidative burst [49,50]. The increase in ROS, in addition to promoting phenolic biosynthesis mediated by PAL activity, also induces phenolic oxidation due to an increase in PPO activity and loss of membrane compartmentalization in a similar fashion as a wound-like effect [51].

Losing selective permeability in the presence of oxygen may lead to membrane leakage and interaction between enzymes (PPO, POD) which are located in the cytoplasm with substrate (phenolic compounds such as hydroxy cinnamic acid and tannins including punicalin, punigalagin in the cells rind vacuole). Higher ratio of PPO/PAL activity is associated with oxidation of phenolic compounds to quinones which creates colored brown products [44]. Eventually, the accumulation of these browning agents in the skin become visible and considered as husk scald.

To avoid this phenomenon of HS in pomegranate during storage various methods were developed in the past as postharvest treatments without knowing the mode of action of HS development. However, we can infer according to the proposed model herein (Figure 3) that the role of these methods is either to inactivate PPO (through limitation of oxygen exposure such as CA, modified atmosphere packaging (MAP), coating or heat) or to avoid contact between the enzyme and its substrate by increasing cell integrity using intermittent warming. 

Investigating postharvest treatments preventing husk scald disorder, is helpful in order to confirm the possible mechanism of HS presented herein. Numerous methods and strategies for postharvest storage of pomegranate avoiding husk scald will be discussed in this review paper and their possible targets.

## 4. Factors Affecting Husk Scald Incidence

### 4.1. Pre-Harvest Factors

Adverse environmental conditions can disrupt normal homeostasis leading to cell death in plant cells and consequently physiological disorder may appear [52]. The tolerance of plant to regulate and maintain cell homeostasis depends on the interaction between genetics and environmental factors. In fact, genetics are a determining factor of plant resistance to physiological disorders [53,54,55]. The information regarding pre-harvest factors affecting husk scald development in pomegranates during storage is limited in the literature.

For instance, the results of a research on seven pomegranate accessions showed that higher antioxidant capacity, total phenolic compound, and punicalin caused lower WL, husk scald incidence and fungi decay in fruit. On the contrary, the amount of punicalagin had not significant correlation with the scald incidence. In addition, anthocyanin content did not show any significant correlation with HS incidence. However, light colored fruit were more sensitive to chilling injury and husk scald, regardless of having high antioxidant activity [40]. Furthermore, neither total soluble solids or total acidity had an effect on HS incidence [40].

There was a correlation between catechol content and peel browning in pomegranate fruit harvested in late season, and there was a stable catechol level in less browned peel during storage [56]. In addition, it has been suggested that pomegranates having a high antioxidant capacity and high total phenolics content in their husks are more tolerant to husk scald and may retard the formation of scalding [15,16,18,40,57,58,59].

Research by [18] showed that late-season harvested pomegranate are more susceptible to HS than mid-season harvested fruits, which is in contrast with the result reported by [13] that indicated that a delay in harvest reduces the incidence of HS. In general, total antioxidant, the level of hydrolysable tannins, such as punicalagin, punicalin and gallagic acid, and total phenols are high in pomegranate skin during the development stage, however, they decrease during the maturation stage and decrease further during storage [59,60]. This might in part explain the reason of higher susceptibility to HS of late-harvested pomegranates. 

**Figure 4:**
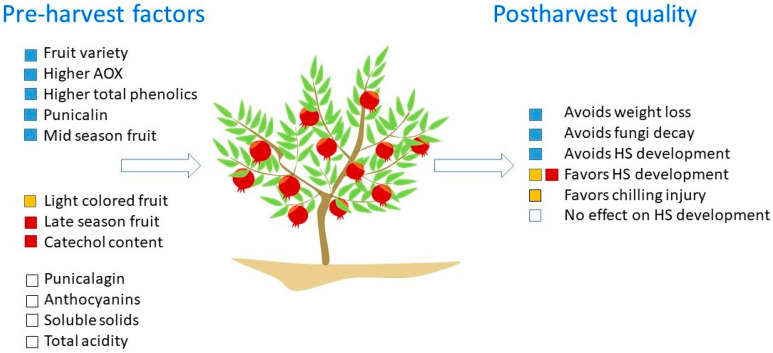
Integral model linking pre-harvest factors and its effects on HS development during postharvest storage. This integrative approach based on reported studied in the literature (see Section 4.1) can be used as a reference for identifying and introducing future studies on pre-harvest factors and also dissect their effects on different pomegranate fruit quality attributes and physiological disorders besides HS (e.g., chilling injury effects).

In Figure 4, we present an integral model based on the above data linking pre-harvest factors and its effects on HS development during storage. This integrative approach may be used as a reference for identifying and introducing future studies on pre-harvest factors and to dissect their effects on different pomegranate fruit quality attributes and physiological disorders besides HS (e.g., chilling injury effects).

### 4.2. Postharvest Treatments for Husk Scald Control

In Table 2, different postharvest treatments and their effects on controlling husk scald incidence in pomegranate fruit are summarized, including the use of CA, MAP, film wrapping, intermittent warming, coatings, exposure to 1-MCP, and the use of exogenous putrescine treatment. In addition, in Figure 3, we present the possible targets of these postharvest treatments based on a model of HS development proposed herein.

#### 4.2.1. Effect of CA

Controlled atmosphere (CA) and modified atmosphere (MA) storage include altering the storage gas environment, specifically the CO_2_ and O_2_ concentrations surrounding the commodities, and can often help to avoid CI or delay the onset of symptoms [25].

Even though pomegranates do not have climacteric respiratory systems, a suitable O_2_ to CO_2_ ratio can prevent peel browning [13]. The ratio has a higher impact on browning avoidance when combined with the proper storage temperature. Previous studies have shown that 2% O_2_, without CO_2_, reduced husk scald and postponed the onset of chilling injury symptoms in Israeli “Wonderful” pomegranate at 2 °C, but husk scald persisted after removal from storage. “Mollar” pomegranates were kept for up to eight weeks at 5 °C and at or above 95% relative humidity (RH) in air and in various combinations of controlled atmospheres, including 10% O_2_ + 5% CO_2_, 5% O_2_ + 5% CO_2_, 5% O_2_ + 10% CO_2_, and 5% O_2_ + 0% CO_2_ ethylene-free. Except at 10% O_2_ + 5% CO_2_, controlled atmospheric storage decreased WL, the risk of decay, and the severity of husk scald.

In contrast to the level previously suggested for the “Wonderful” cv., the “Mollar” cv. appears to be more vulnerable to husk scald than other types. Different cultivars may exhibit varying sensitivity to this disorder [47]. Higher CO_2_ content had a stronger effect in preventing the rate and severity of husk scald after 5 months of storage at 6 °C [48]. The use of CA storage, which combines 5% O_2_ + 15% CO_2_, has the highest success rate for decreasing decay and husk scald problems of all of these methods, it has been shown to prolong the postharvest life of pomegranates for up to 5 months at 7 °C [6]. The increased accumulation of acetaldehyde, ethyl-acetate, and ethanol brought on by anaerobic respiration during CA storage is a drawback because these compounds alter the fruit’s flavor [6,13,18]. Furthermore, CA with less oxygen may induce the formation of ethanol reduce the fruit’s marketability [13]. On the other hand, reduced anthocyanin content, which affects the color of the arils, is a disadvantage of storing with elevated CO_2_ levels [61].

Browning reduction may relate to increased membrane integrity and decreased peroxidase (POD) and polyphenol oxidase (PPO) enzyme activity [62]. Enzymatic browning occurs when various enzymes such as POD and PPO and their substrates such as phenolics, decompartmentalize due to a decrease in membrane integrity [63].

It was shown that loss of phospholipid content in pomegranate skin cells that were stored in air was lower than CA-stored fruit. However, lipophilic conjugates of *p*-coumaric acid (LPCAC) isolated from the neutral lipids fraction of peel tissue lipid extracts in air-stored fruit increased nearly three-fold than CA-stored fruit [18]. LPCAC are known as cutin- or suberin-like oligomers which increase in response of oxidative stress or water loss [64]. Furthermore, hydroxycinnamic acid synthesis may contribute to the browning events involved in scald formation [13].

In general, these results are in agreement with the proposed model of HS development presented herein (Figure 3), where water stress and oxidative stress are key factors in HS development in pomegranate fruit. It is likely that CA reduces oxidative stress when exposed to low levels of oxygen thus reducing HS development.

#### 4.2.2. Effect of MAP

It has been shown that modified atmosphere packaging (MAP) can increase storage life by three months or more and prevent water loss, visible shriveling symptoms, husk scald, and rot in ‘Mollar de Elche’ [10], ‘Ganesh’ [65], ‘Primosole’ [2], ‘Wonderful’ [66], ‘Hicrannar’ and ‘Hicaznar’ [67,68,69] pomegranates.

MAP started to be used frequently for pomegranate shipping and storage. The effect of MAP pomegranate was studied using unperforated polypropylene (UPP) film of of 25 μm thickness and Perforated polypropylene (PPP) film of 20 μm thickness in 2 °C and 5 °C storage.

PPP at 5 °C was the best treatment for maintaining red skin-color of the arils at the end of storage, however, husk-scald development was higher at 5 °C possibly because of an increase in polyphenol oxidase activity. Additionally, only unpackaged control at 5 °C fruits showed a moderate (commercially objectionable) level of pitting and husk-scald severity index [10]. 

Although there are several studies on the effectiveness of MAP for prevention of HS, there is not any information about antioxidants, phenolic compounds or anthocyanin alteration in the fruit skin during storage under MAP treatment. However, in litchi fruit, MAP reduced skin browning through a reduction in PPO and POD activities [70]. Browning of the skin in litchi during storage is the major issue that limits the marketability, the loss of membrane integrity and oxidative stress, which induces skin browning [71]. Higher CO_2_ concentration in MAP has been found effective in maintaining higher antioxidant activity enzymes including CAT, SOD and APX [72], on the other hand due to lower O_2_ concentration, POD and PPO do not catalyze phenols oxidation [70]. Based on our assumption, MAP may have the same effect in pomegranate skin as it has in litchi fruit skin. Furthermore, it has been reported that litchi pericarp laccases play important roles in browning [73] and since laccase and PPO may share common substrate, such as catechol, studies are suggested to determine if laccase activity plays any role in pomegranate fruit HS development. 

It is likely that MAP systems may operate by different modes of action against HS, likely through reduction in water stress and the corresponding oxidative stress signals, and simultaneously by reducing oxygen levels that reduces oxidative stress within the cells (Figure 3). 

#### 4.2.3. Film Wrapping

Pomegranates were preserved at 8 °C for 6 or 12 weeks, and a combination of film wrapping and 600 mgL^−1^ fludioxonil was utilized [74]. The results demonstrated that wrapping could increase marketability, almost entirely prevent husk scald or browning discoloration, and maintain fruit freshness during the entire storage period. Despite the fact that wrapped fruits had higher respiration rates than controls, film wrapping had a significant impact in reducing water loss. According to the model presented herein, this film wrapping likely reduced water loss and did not allow the accumulation of oxidative stress signaling preventing HS development (Figure 3).

#### 4.2.4. Effect of Intermittent Warming

Intermittent warming is the interruption of low-temperature storage with one or more short periods of warm temperature for various periods of time. It has been shown to be beneficial in reducing CI and improving in keeping quality of several horticultural crops, presumably by allowing the continuation of aspects of ripening that had been halted by the low temperature storage [75]. Intermittent warming alleviates husk scald in pomegranates [76]. In chilling injury, intermittent warming has been associated with an increase in unsaturated fatty acid content of membranes increasing fluidity and to an increase in antioxidant levels due to an increment of polyamines that may protect membranes through antioxidant effects [77]. 

Ref. [78] compared pomegranate intermittent warming cycles of 1 day at 20 °C every 6 days, while the fruit was stored at 0 °C or 5 °C. The results showed that storage at 0 °C with intermittent warming avoided decay although increased the risk of chilling injuries such us pitting and husk scald; on the other hand, 5 °C storage with intermittent warming reduced these injuries but fungal attacks were not inhibited.

The application of intermittent warming for 24 h at 15 ± 0.5 °C every 5 days for pomegranate fruit, stored at 5.0 ± 0.5 °C and 5.0% CO_2_ + 8.0% O_2_ could delay browning, with the browning index being 0.15 after 120 days of storage. The results showed that the activities of the browning index of peel were correlated positively with ascorbic acid oxidase (AAO), PPO and POD, while correlated negatively with catalase (CAT) activity. The application of intermittent warming and pomegranate fruit storage under the conditions of 5% CO_2_ + 8% O_2_ gas component could delay peel browning [19].

Although the mode of action of intermittent warming is not clear in HS, it is possible that different mode of actions might be taking place; on the one hand, changes in membrane fluidity and reduced oxidative stress may be favored [77] or alternatively the proposed mechanism of heat shock treatments might take place involving a hierarchical response to stresses, where heat shock-proteins synthesis is favored to a heat stress compared to a subsequent water stress exposure [79]. Thus, according to the proposed model herein, intermittent warming will favor membrane fluidity an antioxidant protection, or may affect proteins synthesized and HS development would not be favored (Figure 3). More research is needed to test this hypothesis of redirecting protein synthesis of key proteins and enzymes due to the application of heat treatments or intermittent warming.

#### 4.2.5. Effect of Coating

Fruit and vegetables are coated to extend their shelf life. Coatings can be made of protein, lipid, polysaccharide, resin, or any combination of, as well as originate from both plant and animal sources. During processing, handling, and storage, coating creates a barrier for gases and moisture and also serve as natural delivery systems for postharvest chemicals [80,81,82,83,84,85,86,87]. Fruit and vegetables can be treated with soy lecithin-based compounds to delay ripening, maintain firmness, reduce physiological problems, and enhance their appearance and marketability [88,89]. The application of commercial formulation of soy lecithin coating on pomegranates significantly reduced fruit WL as well as incidence and severity of husk scald in ‘Primosole’ cultivar. Lecithin slightly decreased the transpiration rate and, therefore, its effect on WL was quite low. It is assumed that the effect of lecithin is because of the antioxidant properties of soy lecithin [75]. The application of MAP and coating with chitosan (CH) alone or in combination decreased HS incidence especially in MAP + CH treatment. MAP and CH + MAP treatments reduced WL by about 5-fold, compared to control treatment while CH coating alone was not effective as much as these treatments in reducing WL during storage and shelf life. Interestingly, CH treatment alone was not as effective as MAP or MAP + CH in controlling HS symptoms [90]. Retention of the husk color in the pomegranate fruit subjected to coating treatments could be attributed to their ability to modify surrounding atmosphere of the fruit, resulting in reduced respiration rate, WL, and inhibited the activity of enzymes that is associated with husk discoloration [91,92]. In general, according to the model presented herein, it is likely that coatings reduce HS incidence by decreasing water loss and avoiding oxidative stress signals (Figure 3).

#### 4.2.6. Effect of 1-MCP

It has been reported that 1-MCP can minimize superficial apple and pear skin scalding and improve the storage and shelf life of fruit [93,94].

Pomegranate is a non-climacteric fruit; studies have shown that some non-climacteric fruits can benefit somewhat from 1-MCP [95,96,97,98]. 

Ref. [18] compared the application of diphenylamine (DPA) and 1-MCP alone and in combination; results showed neither diphenylamine at 1100 or 2200 μLL^−1^, nor 1-methylcyclopropene at 1 μLL^−1^, alone or together reduced scald incidence and severity in pomegranates. On the contrary, it has been reported that the peel browning of ‘Dahongpao’ pomegranates was significantly reduced using 0.25, 0.5, and 1 μL/l 1-MCP concentrations, possibly related to a reduction in PPO activity [99]. Moreover, [100] examined an experiment using 1-MCP in combination with CA (2% O_2_ + 5% CO_2_) and air on “Wonderful” pomegranate. The results showed that 1-MCP can prevent husk scald in pomegranates stored in air storage. The partial control or delay of scald symptoms by 1-MCP shows that the biochemical mechanism(s) causing scald are activated, but not entirely by ethylene [101,102]. Furthermore, 1-MCP and DPA do not effectively prevent superficial scald in pomegranates as they do in apples [41,103].

Even though non-climacteric fruit do not even actually ripen, the low levels of ethylene they release are likely involved in the fruit’s senescence, therefore treating them with 1-MCP may have some advantages. Pomegranates may benefit from lower husk scald and superior aril appearance and taste due to an inhibitory influence on the fruit’s metabolic activity, which leads to senescence. More work has to be conducted to elucidate the role of ethylene in HS development before promoting 1-MCP to control HS development.

#### 4.2.7. Exogenous Putrescine Treatment

Polyamines (PAs) are compounds naturally synthesized in plants and have roles in development, ripening and senescence processes. Putrescine (PUT), spermine (SPE), and spermidine (SPD) are common PAs applied externally to increase the shelf-life of fruit [104]. The application of 2 and 3 mmol/L putrescine before 4 months storage at 5C and 95% RH was effective for husk scald prevention for the first 3 months storage. However, all of the fruit developed scald at the end of 4th month of storage. Moreover, a higher PUT concentration showed a significant decrease in WL of fruit due to maintenance of cell integrity and permeability. Despite the fact that PUT is considered to have antioxidant properties, in this study the treatment was not applied as a coating and thus, there was enough oxygen supply for enzymatic oxidation of phenolic compounds during long-term storage [105]. In general, more work is needed to elucidate any possible role of polyamines in reducing HS incidence and their mode of action.

**Table 2:** Summary of postharvest treatments applied to prevent husk scald (HS) in pomegranate fruit during storage.

Reference	Key Findings	Treatment Description	Fruit Treatment
[13]	Reduced HS	2% O_2_ + 0%CO_2_	**CA**
[47]	The more O_2_ available the more HS occurs	10% O_2_ + 5% CO_2_	
		5% O_2_ + 5% CO_2_	
		5% O_2_ + 0% CO_2_	
[6]	The best combination controlling HS	5% O_2_ + 15% CO_2_	
[48]	The higher CO_2_ concentration The better HS control	3% and 6% CO_2_	
[10]	PPP film and storage at 5 °C the best for controlling HS	Unperforated polypropylene (UPP) film and Perforated polypropylene (PPP) film	**MAP**
[2]	Inhibited HS	Film wrapping in combination with 600 mgL^−1^ fludioxonil	
[78]	Decreased HS	Intermittent warming cycles of 1 day at 20 °C every 6 days	**Intermittent warming**
[19]	Delay HS incidence	Intermittent warming for 1 day at 15 °C every 5 days and 5% CO_2_ + 8% O_2_	
[75]	Reduce severity of HS	Soy lecithin	**Coating**
[90]	Successfully controlled HS	Chitosan treatment 1% with MAP	
[18]	No effect on controlling HS	1-MCP alone or in combination with diphenylamine (DPA)	**1-MCP**
[99]	Peel browning index decreased in all treatments, up to 35% at 0.5 μL/L concentration	1-MCP (0.25, 0.5, and 1 μL/L)	
[100]	1-MCP partially prevents HS specially in air storage	1-MCP combined with controlled atmosphere (2% O_2_ + 5% CO_2_) and air	
[105]	Effective for husk scald prevention for the first 3 months storage, afterwards not effective	2 and 3 mmol/L before storage at 5 °C	**Putrescine**

## 5. Conclusions

Pomegranate is an exotic fruit with high valuable nutritional benefits. This fruit even has the potential to be considered a functional or medicinal food that recently has received much attention among consumers. Due to increased consumptions and demand, extending the marketing season, creating the opportunity for the sustainable consumption of fresh pomegranate and/or its availability to the processing industry by prolonging the storage life of pomegranates is crucial. HS, on the other hand, has a significant negative effect on pomegranate marketability. The results of this review paper and the hypothetical model described herein suggest that storage duration more likely triggers a cascade of oxidative changes that eventually cause a wound-like effect within skin cells and leads to skin enzymatic browning. This process of HS development apparently starts with water loss and a sequential oxidative stress mechanism. Further studies are needed to confirm water loss as the triggering mechanism of HS development. It is interesting that all of the successful postharvest treatments proposed so far in the literature to halt HS development can be explained by the proposed model of HS development described herein, either through control of water loss, control of oxidative stress, maintaining membrane integrity or through redirecting of protein synthesis of key proteins/enzymes involved in HS development. Furthermore, studies are needed to determine the specific phenolics [39] involved in HS development, since the type of phenolic has been shown to influence the hue of the browning polymerization process [106].

This review paper on HS development and mode of action can be the basis to revisit known postharvest treatments and to find and develop new techniques that can prevents HS development. For instance, the combination of treatments using a hurdle approach based on mode of actions of individual treatments may allow us to obtain additive or synergistic effects on the reduction in HS incidence. In spite of the fact that there are a number of studies controlling HS in the literature, proposing a model describing scald etiology and its prevention or even prediction seems essential for developing novel approaches to reduce HS.

## Figures and Tables

**Table 1:** Difference between husk scald and chilling injury in pomegranate fruit during storage.

Husk Scald (HS)	Chilling Injury (CI)
Long-term storage > 10–12 weeks	Short-term storage (starting from 6–8 weeks)
Storage between 6–10 °C or above	Storage < 5 °C
Loss of red pigment and brownish appearance on the stem end that spreads towards the blossom end and peel hardening	Dark brown region scatters in whole fruit skin, pitting
No injury to the arils or locular septa	Affects internal parts and arils

## Data Availability

The data presented in this study are available on request from the corresponding author.
